# Native Entomophages in the Regulation *Cameraria ohridella* Deschka & Dimic Populations: Potential and Limitations for Control

**DOI:** 10.3390/biology15201854

**Published:** 2026-10-15

**Authors:** Liliya R. Bogoutdinova, Ekaterina M. Savenko, Alexander A. Gulevich, Ekaterina N. Baranova

**Affiliations:** 1 N.V. Tsitsin Main Botanical Garden of Russian Academy of Sciences, Botanicheskaya 4, Moscow 127276, Russia; kateryna1900@gmail.com; 2 All-Russia Research Institute of Agricultural Biotechnology, Timiryazevskaya 42, Moscow 127550, Russia; a_gulevich@mail.ru

**Keywords:** *Cameraria ohridella*, parasitoids, entomophages, horse chestnut, monitoring

## Abstract

*Cameraria ohridella* Deschka & Dimić was recorded from Macedonia in the 1980s (Deschka G, Dimić N (1986)). The primary host plant is *Aesculus hippocastanum*. In Russia, this pest was first discovered in 2003 in Kaliningrad. Since the first introduction of *C. ohridella* into Europe, many studies have been conducted on *C. ohridella* parasitoids to identify natural control factors. In this review, we examine the spread of invasive insects identified in recent years, particularly Ohrid horse chestnut leaf miner (*Cameraria ohridella* Deschka & Dimić), and consider the strategies used by native parasitoids to defeat this phytophage. The review also discusses entomophages that feed on *C. ohridella*. Studies show that in Europe, *C. ohridella* is attacked by a diverse range of parasitoids—predominantly native polyphagous *Hymenoptera*, with the family *Eulophidae* predominating. The most significant species are *M. frontalis*, *P. saulius*, *C. trifasciatus*, and *P. agraules*. Significant regional differences in recorded parasitism levels—ranging from 1% to 33.6%—have been identified; these may be attributed not only to biological and ecological factors (such as host developmental stage preferences, as well as spatial, climatic, and phenological conditions) but also to the calculation methodology. The low effectiveness of the *C. ohridella* parasitoid complex in Europe is largely due to poor developmental synchrony between the parasitoids and their hosts. The composition of the assemblage in urban plantings is also influenced by habitat type and anthropogenic disturbance, particularly the removal of fallen leaves. Finally, we provide an overview of future research prospects, with an emphasis on the influence of parasitoids on *C. ohridella* population densities.

## Simple Summary

Over the past ten years, *Cameraria ohridella* Deschka & Dimic (Lepidoptera: Gracillariidae), a leaf miner of the horse chestnut *Aesculus hippocastanum* L., has rapidly spread across most of Europe. Although the use of parasitoids offers potential as a biological control method, a number of issues arise that require detailed study of the interactions and contributions of the predators, parasitoids, and pathogens affecting the horse chestnut leaf miner *C. ohridella*.

## 1. Introduction

Forests are home to a variety of introduced insect pests that affect the structure and function of forests and other green spaces [1] and often cause significant ecological and economic damage [2,3]. Mass outbreaks of pests reduce the viability of trees and can lead to their mass mortality [4,5]. Among invasive phytophagous insects, leaf miners are an important group because many of them are pests of agricultural crops, forest and urban plants [6,7].

The dangerous leaf miner species, *Cameraria ohridella* Deschka & Dimić, has spread across much of Europe and is now found from the UK to western Russia and from Finland to Italy and Spain [8]. It causes significant damage to some species of the genus *Aesculus* and minor damage to species of the genus *Acer* [9,10,11]. Most often, *C. ohridella* develops two or three generations per year and overwinters in the pupal stage in fallen leaves. Multigenerational reproduction, high fecundity and low pressure from natural enemies may explain the maintenance of high population numbers in Europe [12]. One way to reduce the damage caused is to remove fallen leaves in the autumn, in which the pest pupa overwinters [13]. In some countries, injection of systemic insecticides is used to protect particularly valuable trees [7,14].

When penetrating into new regions, leaf miners often become controlled over time by local parasitoids and other natural enemies [7]. *C. ohridella*, as an invasive species, has become a host for native predators [15], pathogens [16], and parasitoids [17,18,19]. However, at present, the combined effect of natural enemies remains insufficient to effectively control *C. ohridella* populations [7].

The aim of this review is to highlight the current problems of the spread of *C. ohridella* in Russia and the regulation of the horse chestnut leaf miner population by a complex of parasitoids.

## 2. Pathways of Distribution of the Horse Chestnut Moth

**Figure 1:**
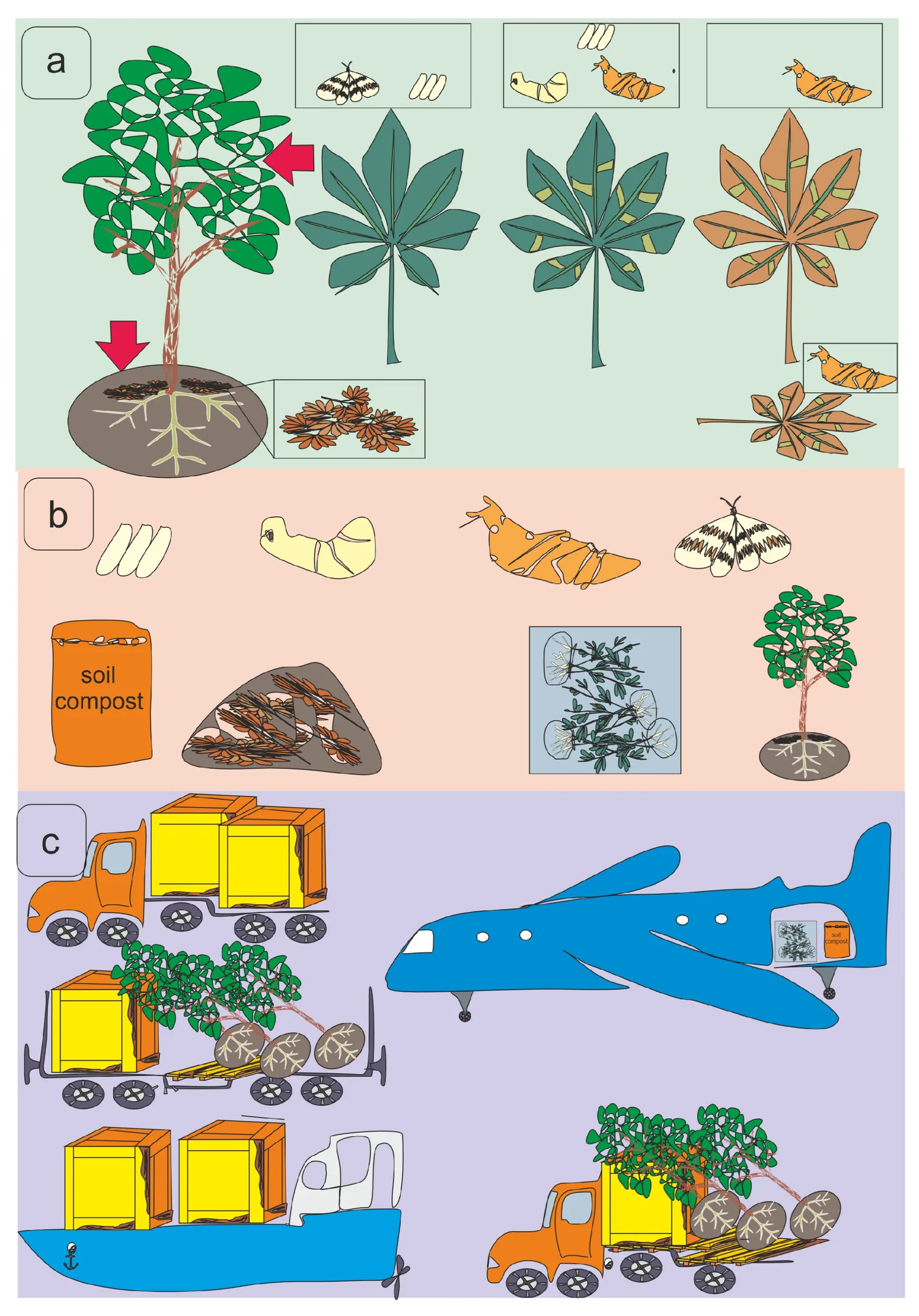
The main pathways for the spread of invasive insects, using the example of different stages of horse chestnut leaf miner development, associated with the logistics of transporting goods, including whole large-sized plants, seedlings, and saplings of horse chestnut, soils and substrates, and products in contact with them during cultivation or transport. (**a**) Locations of detection and probable presence of invasive insect species; (**b**) Movement as laid eggs, larvae of various ages, pupae, or adults during the transport of planting material, infested leaf fragments, wood debris, and containers in vehicles; (**c**) Modes of transport most often involved in the movement of overwintering or dormant invasive insect species in fallen leaves, plant debris, plant fragments, or packaging materials: species can also be transported by transport or in containers or substrates during the transport of live plants.

**Figure 2:**
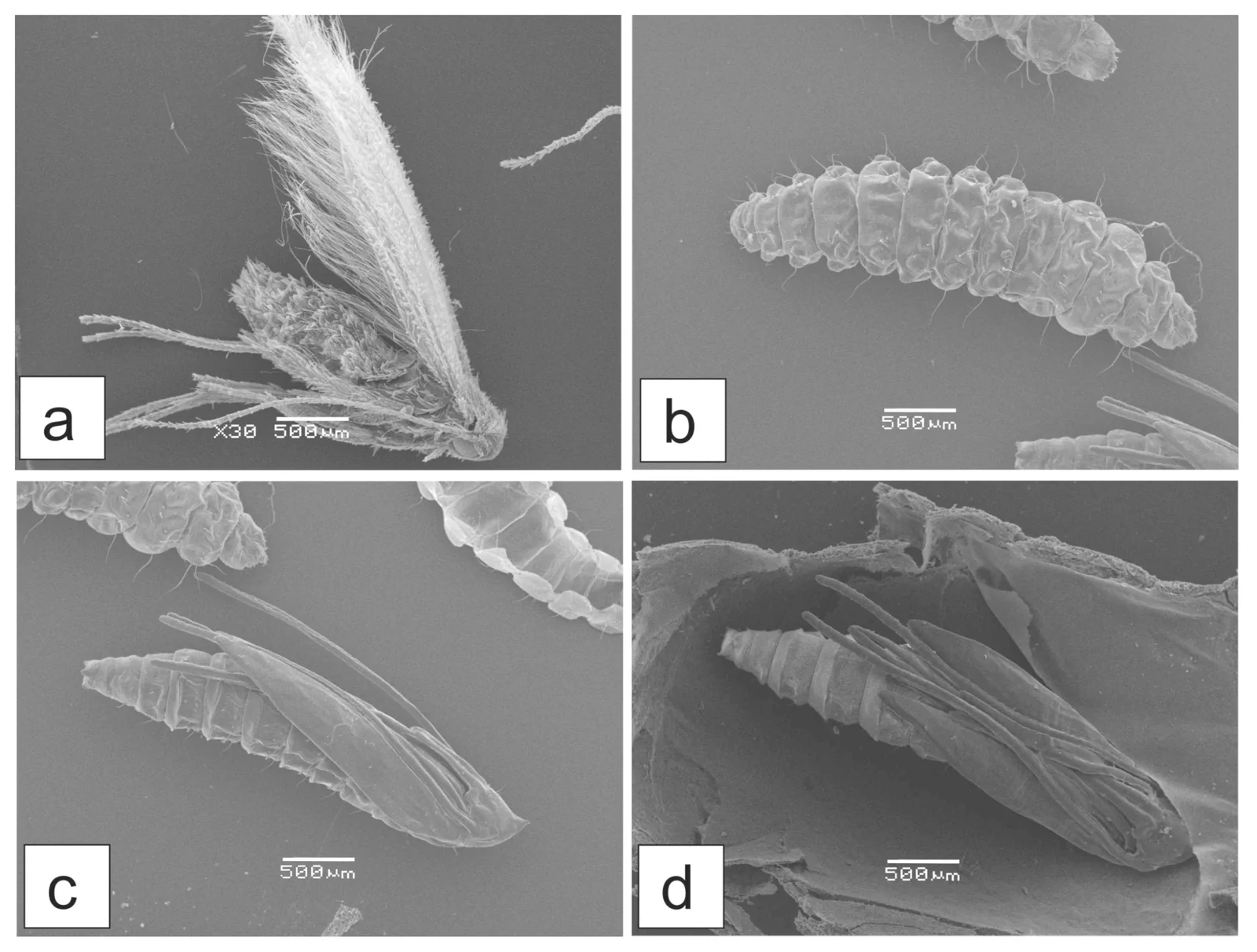
Scanning microscopy of the horse chestnut leaf miner: (**a**) imago, (**b**) older larva, (**c**) pupa, and (**d**) pupa in mine.

Insects that can have a negative impact on tree stands can effectively and uncontrollably cross borders, be transported over significant distances, and subsequently cause significant damage and a significant increase in the costs of restoring tree stands (Figure 1). Eggs, larvae, pupae and adults can be either in a dormant stage or actively developing and moving both on the plant and in the form of plant material, compost, plant parts or seedlings, in containers or timber (Figure 1b). Transport modes can vary widely (Figure 2c); and in modern conditions, control over movements is limited by large volumes of freight transport.

Invasive leaf miners affecting woody plants have had an impact on both the economy and local fauna. For example, in the early 2000s, the control of *C. ohridella* in Germany cost €10.0–33.8 million per year, and the cost of replacing all horse chestnut trees could reach €10.7 billion [20]. The rate of spread of *C. ohridella* in Central and Western Europe is quite significant, according to preliminary estimates based on the first observation data in European countries [21,22]. The biological dispersal of *C. ohridella* occurs according to a stratified dispersal pattern, allowing both long-distance dispersal (wind, human transport) and short-distance dispersal by flight. This type of dispersal is now considered normal for invasive organisms and has been described in other *Gracillariidae*. Local density of population correlates with long-distance spread because transport activity is more intense in densely populated areas. The best model for describing the spread of *C. ohridella* in Germany was a stratified dispersal model that takes into account population density. It outperformed the Gaussian, leptokurtic and stratified settlement models in its predictive ability and spatial configuration of captured territories [23].

In cities, the moth is thought to spread both through wind-blown leaves and through active flight, and indirect observations indicate a spread rate of several hundred meters per generation [24]. Over long distances, *C. ohridella* is presumably spread passively by wind or unintentionally by cars, trucks or trains, as initial habitats are often observed near parking lots and, accidentally, along with infected seedlings in nurseries [22].

## 3. *C. ohridella* and Its Expansion

*C. ohridella* was described in Macedonia in the 1980s [25,26]. It was first recorded in Europe in 1985, in the Lake Ohrid region of Greece. The exact causes of this pathogenicity remain unclear, as studies of herbarium specimens did not reveal the presence of the pest until the last decades of the 20th century. Factors affecting the susceptibility of chestnut to *C. ohridella* are highly diverse. For instance, the effects of tree age, location, light conditions and temperature have been documented [27,28,29,30]. Various microscopy methods, including scanning electron microscopy, are useful for studying the structural features and damage of the *C. ohridella* at different stages of development (Figure 2 and Figure S1).

Different species of horse chestnut are also damaged differently, which is due to the structural features of the leaf blade and the composition of metabolites [31,32,33,34].

It is currently assumed that the routes of emergence of this invasive pest may be associated with the development of logistical schemes and the targeted use of large-sized trees grown in nurseries for urban landscaping (Figure S2). Previously created plantings based on natural introduction from seeds or seedlings with an open root system were damaged for the first time precisely after the start of mass imports of large-sized material (Figure S2a,b). In this case, one can assume both the accidental import of the pest with litter, with overwintering pupae, not even necessarily during the transportation of horse chestnuts, and the import of the pest on leaves in the form of eggs, larvae or pupae, directly in the crown (Figure S2c).

Gradually spreading across the European continent, *C. ohridella* also appeared in the European part of Russia [11,17]. In Russia, its first discovery dates back to 2003 and was in the city of Kaliningrad (southeastern coast of the Baltic Sea). In 2005, the leaf miner was registered in Moscow [35], approximately 1000 km east of Kaliningrad. In subsequent years, this species was discovered in a number of regions of the European part of Russia, where horse chestnut is grown as a popular ornamental tree [36]. By 2015, *C. ohridella* was recorded in 13 central regions of European Russia [37]. In the European part of Russia, *C. ohridella* develops from early May to early October and produces two to three generations per year. In Moscow and Pskov it develops in two generations, and the third generation is usually incomplete [38,39]. In the south, it is capable of producing four generations per year depending on climate changes and average temperature [40,41].

The main host plant is the horse chestnut, *Aesculus hippocastanum* L., a species native to the Balkans that began to be widely planted in Europe in the early 17th century [42]. *C. ohridella* mines were also found on *Acer platanoides* L. in the N.V. Tsitsin Main Botanical Garden of the Russian Academy of Sciences (Moscow) in 2006–2009, but the larvae failed to pupate. In Moscow it can successfully develop on *Aesculus glabra* Willd [38].

Since 2014, *C. ohridella* has become noticeable in the parks of St. Petersburg [25]. The average number of mines/caterpillars, even in the first generation, can reach 175 on one compound leaf, which is why by the end of June the leaves of the horse chestnut turn brown and begin to fall [42]. In 2018, in St. Petersburg, despite the low density of the first generation, the proportion of mined leaves in the second generation already amounted to 90–100%, which was probably due to favorable weather conditions [25].

Similar to European countries, where *C. ohridella* causes severe aesthetic damage to horse chestnut trees [8], the horse chestnut leaf miner is one of the dangerous pests of *Aesculus hippocastanum* in the European part of Russia [38,43]. It is one of the most dangerous invasive insect species in Russia [44].

## 4. Methods of Control of *C. ohridella*

Several methods are used to combat the horse chestnut leaf miner. High mortality rates of the pest’s larvae are achieved with the use of systemic insecticides, but spraying is expensive and difficult to implement in urban areas [45] or is generally prohibited for use in urban areas [38]. However, positive results were demonstrated using the stem injection method. A two-year trial was conducted in Moscow on horse chestnut trees using abazol stem injection, which demonstrated high efficacy the following year. Another effective control method is the complete removal of fallen leaves in which the pupae overwinter. In the European part of Russia, pheromone traps with the synthetic pheromone *C. ohridella* were used to study the population dynamics of the miner [46]. Unitrap pheromone traps and Delta sticky traps have shown high efficiency in monitoring *C. ohridella* [34]. Methods of biological control, such as the release of specialized parasitoids of the leaf miner, are still far from practical application in the European part of Russia [38]. One way to reduce the damage caused by leaf miners is to use species in landscaping that are less susceptible to these pests [47]. Thus, in European parks, *Aesculus carnea* Hayne, a hybrid between *A. hippocastanum* and *Aesculus pavia* L. is used, on the leaves of which the caterpillars of the leaf miner die in the early stages of development before they begin feeding on the palisade tissue. If left without a choice, the horse chestnut leaf miner can develop on *Aesculus hippocastanum*, *Aesculus turbinata* Blume, *Aesculus flava* Solander and *Aesculus pavia*, *Aesculus parviflora* Walt., *Aesculus carnea*, *Aesculus glabra* Willd. and *Aesculus indica* Colebr. [47] turned out to be resistant to damage by the horse chestnut leaf miner, but on *Aesculus chinensis* it does not complete development to imago. However, it has been estimated that, for example, replacing Berlin horse chestnuts with resistant species would require around 300 million € [48].

## 5. Natural Enemies of *C. ohridella*

An important role is played by natural enemies of miners: parasitoids, predators and pathogenic microorganisms. Among invertebrates, ants most actively eat leaf miner moths. Thus, in forest park conditions, *Formica rufa* L. ants can collect almost all the pupae of *Gracillaria syringella* F. from the litter at a depth of up to 2 cm [49]. *Formica uralensis* Ruzsk. Ants collected larvae of the moth *Ocnerostoma friesei* Svensson within a radius of 60 m from the anthill [50]. Observations exist of successful predation by *Crematogaster scutellaris* on *C. ohridella* [51].

It has been experimentally demonstrated that *Meconema meridionale* Costa locates larvae within mines and gnaws out them from the leaves, consuming on average up to two-thirds of all larvae on a single leaf per day. The authors also hypothesized that *M. meridionale* might influence the abundance of the leaf-mining moth. However, the effectiveness of this impact may remain negligible due to the low population density of *M. meridionale* on the trees and its polyphagous nature. In turn, artificially increasing the *M. meridionale* population may be inadvisable, as this could have unforeseen ecological impacts. Since *M. meridionale* inhabits trees that are frequently infested by other leaf-mining moths, it is possible that it preys on leaf-mining moths in general [15].

The egg is the most vulnerable stage of the moth’s development on the surface. However, experiments have shown that the common omnivorous predatory mite *Euseius finlandicus* Oudemans does not prey on *C. ohridella* eggs, as none of the individuals consumed a single egg. It was hypothesized that *E. finlandicus* does not find *C. ohridella* eggs [15].

The main predators of life miner moths are insectivorous birds. Thus, tits of the genus *Parus* are considered the main predators of the moth *Cameraria hamadryadella* Clemens [52]. The same was found in a study of the predator complex in the secondary habitat of *C. ohridella* [15]. In this case, tits destroyed from 2 to 4% of the moth population. However, in urban areas, they also feed on other insects, spiders, and bird food [53]. In this context, volatile substances released by plants when attacked by herbivorous insects play an important role in prey detection by insectivorous birds [54]. The absence of birds can lead to a significant increase in the colonization of host plants by leaf miners. Tits feed on older butterfly caterpillars or pupae and peck at mines from above [15], while insects often chew holes in the underside of mines (e.g., *Meconema meridionale*) [7].

Birds not only hunt large *C. ohridella* individuals but also open mines, thereby largely preventing damage to trees. Thus, attracting birds, for example by installing birdhouses, is one of the methods of comprehensive prevention that can significantly help in protecting plants. The most important factor is the size of the entrance hole in the birdhouse, which promotes breeding of birds such as blue tits and great tits [55].

It has been hypothesized that birds preying on herbivorous insects may either consume parasitoid insects or reduce their effectiveness through competition for the same prey [56]. However, parasitoid wasps are too small to serve as prey for blue tits or great tits [53]. Nevertheless, parasitoid wasps must first reach a sufficient population density to reduce the numbers of leaf-mining moths; furthermore, due to their small size and predominantly passive movement, these parasitoids may be more sensitive to climatic factors and fluctuations in host population density than birds [55].

Well-known entomopathogenic fungi (*Beauveria* Vuill., *Lecanicillium* W. Gams and Zare, *Metarhizium* Sorokin, and *Paecilomyces* Bainier) were isolated from the habitat of C. ohridella, and some strains demonstrated entomopathogenic activity against the moth’s pupae and caterpillars [57,58]. Information on the virulence of fungi against all developmental stages of the moth remains insufficient. In a laboratory study conducted in Lithuania, eggs, larvae, and pupae were transferred from their natural environment to laboratory conditions favorable for fungal development. The results showed that both overall mortality and mortality associated with mycoses were lower than in the wild [16]. There is no doubt that the probability of fungal infection would have increased with prolonged exposure.

Twenty-seven species belonging to 19 genera were identified, including eight species of entomopathogenic fungi, seven species of secondary fungal pathogens, and 12 species of saprotrophic fungi. Different types of fungi predominated depending on the moth’s developmental stage. At the egg stage, *Cladosporium cladosporioides* (30.68% of all mycoses) primarily caused the mycoses; at the pupal stage, *Cordyceps fumosorosea* and *Beauveria bassiana* predominated (17.49% and 16.94%, respectively); and at the adult stage, *Lecanicillium aphanoclaii* was the most prevalent (39.45%). However, there is very little information on the diversity of entomopathogenic fungi responsible for *C. ohridella* mortality [16].

## 6. Parasitoids of the Horse Chestnut Leaf Miner

### 6.1. Species Composition of Parasitoids

Since the first invasion of *C. ohridella* in Europe, many studies have been carried out on *C. ohridella* parasitoids in order to identify natural control factors [59,60,61]. There is evidence that leaf miner parasitoids search for suitable host trees rather than specific insect hosts [62]. In addition, toxic substances from *A. hippocastanum* consumed by leaf miner larvae may protect them from attack by natural enemies. In addition, the phenology of parasitoids is not synchronized with the development of miners in the spring, which may hinder parasitism in the first generation [63]. However, the location height of the mines at different tree tiers does not appear to affect parasitism. Parasitism rates did not vary with the height of the leaf miners, although several studies indicate that leaf miner numbers are higher in lower parts of the canopy [64].

In the secondary range, the leaf miner is attacked by a complex of local entomophages [65,66], and the species complex of parasitoids in the invasive foci turned out to be generally identical to the natural one [67]. These parasitoids are mainly broadly polyphagous, while mono- and oligophages are absent. Seven species of local polyphagous insects were identified, with *Minotetrastichus frontalis* L., *Closterocerus trifasciatus* Westw. and *Colastes braconius* Haliday predominating. Sixteen species of parasitoids of horse chestnut leaf miner are known in Poland, and more than 30 species in Macedonia, but they destroy only 1% of individuals [68]. In the Balkans, the parasitoid complex of the horse chestnut leaf miner is dominated by *M. frontalis* and *Pediobius saulius* (Walker) [26]. Thus, the parasitoid species found in the Czech Republic are similar to those found in Bulgaria, Greece, Croatia, Macedonia and Yugoslavia, or in South-Eastern Europe in general [17]. In Japan, two species of the *Eulophidae* family were found among the parasitoids of linden leaf miners; in Europe, six; and in the Volga region, twelve [59].

In Europe, parasitoids of *C. ohridella* belonging to the families *Braconidae*, *Ichneumonidae*, *Pteromalidae*, *Eupelmidae* and *Eulophidae* have been recorded; however, the most widely represented representatives are the family *Eulophidae* [59,69]. As a result, a list of entomophages of the horse chestnut leaf miner was compiled based on data from 23 European countries (Russia, Belgium, Austria, Greece, Bulgaria, Macedonia, Croatia, Serbia, Switzerland, Ukraine, Slovakia, Slovenia, Turkey, Hungary, Poland, Sweden, the Czech Republic, Italy, Germany, Bosnia and Herzegovina, France, Romania and the United Kingdom). In the structure of the entomophagous complex of *C. ohridella* in the fauna of Europe, more than 73% are representatives of the *Eulophidae* family, while the rest of the *Hymenoptera* family account for a total of 27%. The most common chalcidoid was *M. frontalis (Eulophidae)*, accounting for 77.5% of all parasitism cases. Other regularly collected parasitoid species include the solitary parasitoids *C. trifasciatus (Eulophidae)* and *Pnigalio agraules* (Walker) (*Eulophidae*). The remaining parasitoids were rare, accounting for less than 1% of the total parasitism. *Minotetrastichus platanellus* (Mercet) (*Eulophidae)* increased its parasitism share from 0.8 to 7.5% since 2003 [58]. In *M. frontalis*, more than one larva was observed on one host. Multiparasitism was sometimes observed between *M. frontalis*/*P. saulius* (*Eulophidae*) and *M. frontalis*/*Closterocetus trifasciatus* (Westwood) [70].

Thus, in Europe, at least 60 species of *C. ohridella* parasitoids are known [71], but the level of parasitism remains generally low, within 10–25% in southeastern Europe and below 10% in European countries recently colonized by the pest [72,73]. In exceptional cases in the southern European part of Russia, particularly in the city of Krasnodar, the parasitism rate reached 33.6% by 2013. This parasitoid complex includes wasps from the *Braconidae*, *Eupelmidae*, *Ichneumonidae*, *Pteromalidae*, and *Eulophidae* (*Hymenoptera*) families, most of which have diverse trophic relationships with local insects. The complex has proven itself as one of the factors in the fight against *C. ohridella* in Krasnodar, significantly reducing the population density by 2014 compared to 2010 [74].

Table 1 provides a list of the most common parasitoids both in Russia and in a number of European countries.

Species parasitizing *C. ohridella* include *Pteromalus semotus*, *Cirrospilus viticola*, *C. vittatus*, *Pnigalio agraules*, *P. pectini—cornis*, *Sympiesis sericeicornis*, *Chrysocharis nephereus*, *Ch. pentheus*, *Closterocerus trifasciatus*, *Pediobius saulius*, *Baryscapus nigroviolaceus*, *Minotetrastichus frontalis*, *Colastes braconius*, *Scambus inanis* [79]. Among them, the most common types are the following:

*Minotetrastichus frontalis* is an ectoparasitoid of caterpillars and pupae of leaf miners. *M. frontalis* overwinters as a larva in mines [18]. The parasitoid attacks leaf miners at stages from young caterpillars (L2) to pupae but prefers older caterpillars (L4), as they provide a larger food source for the parasitoid larvae [70]. *M. frontalis* larvae were found. In a number of cases, *M. frontalis* was noted as a hyperparasitoid; cases of multiparasitism of *M. frontalis* with *P. saulius* and *C. trifasciatus* are also known [59]. In cases where a wasp of another species of eulophid is already developing inside the mine on the host, and the host’s food resource has been used up, the females infect the larvae or pupae of the primary parasite, and the *M. frontalis* larvae develop on it as secondary parasites [80].

*Pediobius saulius* is a primary, often secondary, rarely tertiary solitary endoparasitoid of larvae and pupae of mainly leaf-mining insects [11]. These are the countries of the Balkan Peninsula and their neighbors—Slovakia [59] and Hungary [70]. The parasitoid attacks the miner from older caterpillars (L4) to the pupal stage, preferring the pupal stage [17]. Cases of hyperparasitism of *P. saulius* on *Colastes braconius* are known. Females infect the larvae or pupae of the primary parasite when the host’s food supply is exhausted.

*C. trifasciatus* is a primary, less commonly secondary, solitary endoparasitoid of larvae and pupae [81]. The larva overwinters inside the host pupa [18]. *C. trifasciatus* is widely known as a parasitoid of *C. ohridella*. *C. trifasciatus* attacks *C. ohridella* from early instar caterpillars (L2) to pupae, preferring late stages of the miner’s development [17]. It is known as a hyperparasitoid of the larvae and pupae of other parasitoids such as *Chrysocharis* spp. [17] and *P. saulius* [82] when the host’s food resource is exhausted.

*Pnigalio agraules* is a solitary ectoparasitoid of mainly leaf miner larvae [81]. One of the important parasitoids, *C. ohridella*, found in 20 countries. The species has been recorded as subdominant in Great Britain [82]. *P. agraules* attacks fourth instar caterpillars [83] of *C. ohridella* [82]. In a number of cases, *P. agraules* was noted as a hyperparasitoid on the pupae of *Eulophidae* [59]. A method for growing *P. agraules* in laboratory conditions has been developed [83].

Studies show that in Europe, *C. ohridella* is attacked by a diverse range of parasitoids—predominantly native polyphagous parasitoids from order *Hymenoptera*, with the *Eulophidae* predominating. The most significant species are *M. frontalis*, *P*. *saulius*, *C. trifasciatus*, and *P. agraules*. At the same time, the parasitoid complex as a whole is similar across different ranges and does not include species that specialize exclusively on *C. ohridella*. The main discrepancy between studies concerns the assessment of the actual regulating role of individual species within the parasitoid complex. Some species are also hyperparasitoids and can attack the larvae of other parasitoids, not just *C. ohridella*. The ecological and biological factors contributing to the shift in parasitism by polyphagous species from other insect species to *C. ohridella* have not been sufficiently studied. Quantifying the species-specific contribution of parasitoids, particularly for common species such as *M. frontalis*, *P. saulius*, *C. trifasciatus*, and *P. agraules*, can be used to effectively suppress leaf miner populations.

### 6.2. Levels of Parasitism in Different Countries and Methodological Differences in Their Assessment

Thus, methods for rearing traditional parasitoid species can be developed that can later be used for biological control, since asynchronous release of parasitoids and *C. ohridella* can be avoided.

Research on parasitoids is typically conducted based on the objectives of specific research projects and the collection and monitoring protocols adopted by research groups; this can create significant challenges when comparing and interpreting results. Establishing common requirements and harmonizing methodology would provide a clear and unambiguous picture—one that currently remains merely approximate (Table 2).

In the Czech Republic, Slovakia, and Slovenia, 500 to 700 mines per sample were dissected to determine the level of parasitism, and the numbers of live, dead, missing, and parasitized larvae or pupae were recorded. Parasitized, dead, weak, or unhealthy larvae, as well as all pupae, were placed individually into plastic Petri dishes. This method is based on integrating data on parasitic infestation and mortality occurring at various developmental stages unrelated to parasitism. It avoids common errors associated with dissection and is independent of the sampling date or the proportion of different developmental stages in the samples, given that the level of parasitism varies significantly depending on the developmental stage. However, to obtain an accurate picture of the proportion of the population that actually perishes due to parasitoids, it is necessary to consider at least two other parameters: host nutrition and other mortality factors, such as those caused by intraspecific competition and leaf senescence in autumn [76].

In London, leaves collected from each tree were used to examine mines and determine reproductive parameters, while two leaves were used to rear parasitoids. Dissection of the mines was the primary method for determining parasitism levels and identifying parasitoids. For each dissected mine, the developmental stage of *C. ohridella* was determined based on a combination of mine size, larval size and coloration, and the number of exuviae found within the mine. The emerged parasitoids were identified based on the presence of a circular hole (1.5 mm or less in diameter) on the upper surface of the leaf or mine, as well as the presence of their exuviae, larvae, or pupae. The higher level of parasitism observed in urban plantings compared to rural ones may be due to the small sample size or the higher temperatures found in urban areas. The zero level of parasitism at Danebury Avenue during the first sampling period can be attributed to the late colonization of the site by *C. ohridella*—a result of local authorities collecting fallen leaves—which provided insufficient resources for the development of parasitoids [78].

Horse chestnut leaf samples (specifically *A. hippocastanum*) infested with *C. ohridella* were collected from six locations representing the main regions of the Czech Republic. The sampled trees were located in urban parks, established avenues, or roadside tree groups with underlying lawns, and were subject to varying levels of maintenance, such as mowing and leaf removal. Leaflets were carefully cut from the compound horse chestnut leaves, and 500 mines per sample were dissected under a stereomicroscope, with a maximum of 10 mines taken from any single leaflet. The numbers of live, dead, and infected larvae and pupae were recorded. Parasitism rates at various developmental stages and other mortality indices were calculated using the method of Volter and Kenis [76]. The observed low rates of parasitism are likely the result of the local parasitoids’ insufficient adaptation to the new host. Differences in parasitism rates between sampling sites may be attributable to varying local climatic conditions and factors related to street and building layouts and the prevailing wind direction. A key factor appears to be the maintenance of public green spaces—specifically the raking and removal of fallen leaves in autumn or early spring—which is a recommended and effective method for managing the damage caused by *C. ohridella* in the spring [70].

In Southeastern Europe, infested horse chestnut leaves were collected when the majority of the moths were in the pupal stage. Leaves with mines were selected at random from the lower branches of the trees, and the contents of 130–700 mines (400–500 mines in most cases) were examined under a stereomicroscope. Quantitative analysis of parasitism was based exclusively on the results of dissections. The percentage of infested mines relative to the total number of opened mines at each site was selected as the measure of parasitism. The polyphagous species *P. saulius* clearly predominates in the majority of samples; its dominance depends on the presence of a significant proportion of leaf-mining moths at the pupal stage. The second dominant species, *M. frontalis*, parasitizes a wide range of leaf-mining moth developmental stages and becomes more significant in samples where pupae are scarce. These differences are most likely attributable to spatial or temporal heterogeneity in the sampling sites and dates. For example, the abundance of *Baryscapus nigroviolaceus* was higher at several sites in Greece, regardless of habitat type [72].

In Switzerland, monitoring was conducted using two methods. The first method involved examining first-generation larvae—specifically those in the first and second developmental stages—at the onset of their development; their position on the leaf was precisely recorded, and a tag was attached to the leaf. The mines were monitored until the emergence of an adult *C. ohridella* or until the larva died or was paralyzed by a parasitoid. The second method involved dissecting the mines every two weeks. Upon discovery of an infected larva or pupa, the mine was placed in a Petri dish until the parasitoid emerged. Parasitism rates were calculated separately for the three butterfly developmental stages most heavily affected [i.e., the final larval stage (L4)]. Calculations were performed using the formula: Parasitism at stage I = number of parasitized hosts at stage I/number of insects (dead or alive) at stage I and subsequent stages (including emerged adults).

The phenology of *C. ohridella* parasitoids is not well synchronized with that of their new host. All parasitoids identified in this study are highly polyphagous. Most parasitoids attacking the first generation of *C. ohridella* in the spring are likely individuals that emerged earlier or later and survived until suitable host developmental stages became available. Overall, a greater number of parasitoid species were found in forest sites, with three species—including *Pnigalio* cf. *soemius* and *Colastes braconius*—detected exclusively in forest areas; this suggests that biologically rich habitats may support a larger number of incidentally encountered parasitoid species. The level of parasitism did not increase from the first to the second generation. A lack of synchronization is likely not the cause of the low level of parasitism observed in the second half of the season, since the generations overlap by that time and suitable host developmental stages can be found at any time. Consequently, there may be physiological or chemical barriers preventing the complete colonization of *C. ohridella* by native parasitoids [19].

Thus, dominant species among the primary parasitoids of *C. ohridella* in a given area may compete with other parasitoid species for their niche and role. Another reason could be the lack of evolutionary associations between secondary parasitoids and the specific “host plant—leaf-mining insect—primary parasitoid” combination. The establishment of new, stable infochemical connections among the parasitoid complex species associated with a new phytophage (and, in our case, on an introduced plant as well) takes time. A common issue affecting the functioning of phytophage parasitoid complexes in Europe is the prevalence of artificial plantings combined with significant anthropogenic pressure. The high population density of the horse-chestnut leaf miner across a vast area of the Balkans, coupled with low parasitism rates, suggests that this region may not be the primary range of *C. ohridella* [75].

The parasitoid complex of *C. ohridella* in various regions of Europe is represented predominantly by polyphagous species, among which *P. saulius* and *M. frontalis* are most frequently recorded. The level of parasitism depends on preferences regarding the host’s developmental stages, as well as spatial, climatic, and phenological conditions. The composition of the complex in urban plantings is also influenced by habitat type and anthropogenic disturbance—specifically, the removal of fallen leaves. Significant differences in the recorded level of parasitism have been observed between regions. This may be attributable not only to the effectiveness of the parasitoids but also to the calculation methodology. Thus, varying sample sizes, sampling times and locations, and developmental stages of *C. ohridella* are used. Detection methods include opening mines, laboratory rearing of parasitoids, and sequential field monitoring. Therefore, differences in parasitism rates between countries may stem from methodological rather than solely biological factors, given the lack of a standardized protocol for assessing parasitism. Conducting standardized monitoring using uniform criteria for material selection is a promising approach.

### 6.3. Phenological Synchrony and Mechanisms of Parasitism

Results of previous studies of *C. ohridella* have revealed signs of poor synchrony between parasitoids and hosts, particularly in spring. The frequency of parasitism of the first generation of leaf miners in spring is consistently low, regardless of the frequency of parasitism of the previous generation in autumn [62,84]. The average parasitism level of the overwintered generation was low—only 2.4, 2.9 and 3.1% in 2003, 2004 and 2005, respectively. Quite low rates of parasitism were also noted in the studies of other authors [9,67,85]. Parasitism rates in pupae ranged from 0 to 12% and from 4 to 28% in Belgrade and Istanbul, respectively [85]. It was found that higher levels of parasitism were observed at later stages [59]. In addition, the complex of parasitoids attacking *C. ohridella* in autumn differs in composition from the complex observed in spring. This suggests that the parasitoids that enter diapause in *C. ohridella* mines in autumn are not the same parasites that infect the first generation of leaf miners in spring. This may be due to the lack of synchronization between the miner and the parasitoids, as well as the fact that many parasitoids are polyphagous.

On the other hand, parasitic wasps are known to be polyphagous [86] and are likely to encounter a variety of other potential host species. Consequently, parasitism of the first generation of *C. ohridella* by native parasitoids starts at the same low level each year. The absence of parasitoids specialized in relation to *C. ohridella* was noted; all identified species are polyphagous, although biotypes specific to the horse chestnut leaf miner cannot be excluded [87]. A tendency towards an increase in the level of parasitism of local parasitoids in invasive foci was revealed in connection with the duration of the invader’s stay in the territory [17]. This may be due to the fact that the parasitoids are from the same region of origin as the host [44].

A highly significant correlation has been shown between parasitoids and host preference for late larval stages and pupae, for example, in *M. frontalis* [63]. Thus, at the end of May, based on the results of the autopsy of the first researched mines, larvae of the third stage of development, suitable for the development of the parasitoid, were discovered [63]. A significant time delay was found between the emergence of parasitoids from horse chestnut leaf litter and the appearance of suitable host developmental stages [86]. Thus, the low level of parasitism in the first generation under artificial colonization by *Minotetrastichus frontalis* Nees may be due to the fact that *M. frontalis* Nees overwinter in dry leaves and leave them when host mines are not yet present [77].

Differences in parasitism rates may be due to different local climatic conditions and factors related to city characteristics. A study of chalcidoid wasps hatched from horse chestnut leaf litter samples collected at 35 sites in the Czech Republic [88] showed that their abundance was negatively correlated with latitude and positively correlated with altitude and with *C. ohridella* abundance. Apparently, removing horse chestnut leaf litter also reduces the number of parasitoids that overwinter in horse chestnut leaf litter [89].

According to Vercher [90], the low parasitism rate may be due to the absence of native species in the new invasive host, but it may also be partly due to density effects, as many parasitoids exhibit negative density dependence at very high host densities. In comparison, the parasitism rate in *C. ohridella* is very low [91]. The highest rates of parasitism were found in Serbia and Macedonia [92] and in Austria [84]. The parasitism rates in *C. ohridella* in Turin were 1.4, 1.8, 9.3, 14.7 and 5.4% over a 5-year period, reaching 40.0% in some samples collected in 2004 in the city park [93].

There is evidence that parasitoids that attack leaf miners search for suitable host trees rather than specific host insects [62]. In addition, toxic substances consumed by leaf miner larvae may protect them from attacks by natural enemies [63]. The level of parasitism does not vary with tree height, although several studies have shown that leaf miner numbers are higher in the lower parts of the crown [94]. Local parasitoid species that attack leaf miners are capable of switching to this new host soon after first being detected in an area, as was observed with the appearance of *C. ohridella* in Turin. It is possible that these parasitoid species attack similar hosts with similar ecology [90]. The low parasitism rate may be due to density effects, as many parasitoids have a negative dependence at high host densities, as local parasitoids need more time to adapt to a new host. Leaf plants with their host diversity help maintain an important parasitoid complex of leaf miner communities that can play an important role in controlling pest species [64].

There is a theory that a number of features in the biology and ecology of *C. ohridella*, such as the ineffectiveness of control by natural enemies, indicate a possible exotic origin of *C. ohridella*. It is likely that this species is not endemic to the Balkans but was introduced to the area of its discovery from another continent [95]. These results suggest that the *C. ohridella* parasitoid complex in natural and artificially created horse chestnut stands in Europe is not as effective for this very reason. The authors consider three possible origins: from *Aesculus hippocastanum* or another *Aesculus* species from another continent, especially in East or Southeast Asia and North America; from Southeast Europe, where it could have changed hosts, as suggested by [86] and appeared at very low density on its original host plant in this region and remained unnoticed; or *C. ohridella* could be from another continent and feed on a different host plant [95].

The low effectiveness of the *C. ohridella* parasitoid complex in Europe is largely due to poor synchrony between parasitoids and hosts, particularly at the beginning of the growing season, as parasitoids often emerge from diapause before the larval stages of *C. ohridella* susceptible to parasitism become available. Other factors include the lack of parasitoid specialization on *C. ohridella* and the dependence of parasitism efficiency on regional climatic conditions. At the same time, it has been shown that the level of parasitism may increase with the duration of *C. ohridella* presence in the area inhabited by the parasitoids. The low level of parasitism may also be explained by possible negative effects associated with high population densities of *C. ohridella*. It is unclear to what extent differences in parasitism levels are driven by density or environmental factors and to what extent by species-specific characteristics of the parasitoid life cycle. Standardized, long-term studies involving the simultaneous monitoring of host and parasitoid development are required.

### 6.4. Behavioral Characteristics and Parasitism Strategies

Determining the location of the host is preceded by complex search behavior of females of the eulophid family, allowing them to determine the location of the host in the thickness of plant tissue. Using an ovipositor, the wasp makes a puncture into the location of the phytophage (ectoparasitism) or into its body (endoparasitism). The criteria for the selection of an oviposition site by female wasps are: the hidden location of the host larva (to protect the parasite’s offspring inside the mines), the size of the host, and the stage of host development (the wasp larvae need a sufficient food resource, especially during the multiple development of several individuals on one host) [96].

Therefore, larger hosts are generally more profitable than smaller ones because they provide more resources [97]. When choosing a host, parasitoids take into account not only the size of the host but also its age or stage of development, since this affects the growth and reproduction of the parasitoid [75]. Hyperparasitism can be read as one of the factors that prevent parasitism. The fact of hyperparasitic development of eulophids indicates a wide trophic specialization of the parasitoid wasps of the studied group and their ability to adapt to hosts of different species, which develop hidden in the tissues of the plant, depending on the circumstances: in the presence of a mine or gall of a phytophage inside, parasitism occurs on it; in the absence of a host, the wasps switch to hyperparasitism, developing on primary parasites of other species of eulophids or on representatives of their own species, which are the offspring of another female (who infected the host earlier) [80].

For eulophids to successfully parasitize a leaf-mining host, the host’s accessibility, size, and developmental stage play a crucial role. For instance, larger host individuals at specific developmental stages generally provide the parasitoid with more resources for offspring development. At the same time, the ability of certain species to shift from primary parasitism to hyperparasitism indicates the absence of a unified host-utilization strategy and suggests that the parasitic mode depends on the availability and quality of the potential resource. However, the extent to which host characteristics and the presence of a primary parasitoid influence the choice between primary parasitism and hyperparasitism remains insufficiently studied. Identifying these patterns can play an important role in understanding the mechanisms underlying the formation of parasitoid communities.

## 7. Study of *C. ohridella* Population Size and the Impact of Parasitoid Number and Other Biotic Factors on It

Sex pheromones have been isolated, identified, and synthesized for species of the *Gracillariidae* family, high doses of which disrupt males’ search for females. Therefore, another method for reducing leaf miner populations is sticky traps with pheromones [98]. Thus, the use of pheromone traps helped to reduce the population density of horse chestnut leaf miner moth, with more moths captured on trunks than in crowns [68]. For *C. ohridella*, the suitability of pheromone traps [99,100] for assessing the level of infestation was assessed by observing leaf damage and counting the number of males caught in pheromone traps baited with *C. ohridella* sex pheromone. For these observations, the damage index was obtained based on a visual assessment of the total proportion of leaves damaged by moths. This scale has been shown to be linearly related to the number of mines at the leaf level [101,102], which, as a result of the direct correlation, allows for the estimation of population size using pheromone traps baited with the sex pheromone of *C. ohridella*.

*C. ohridella* population density on horse chestnuts can be estimated independently based on observations of both foliar damage and pheromone trap catch data. In Brussels, a fairly strong linear relationship was found between log-transformed data obtained using first-generation pheromone traps and subsequent infestation events observed during the survey. In France, correlations between damage rates and log-transformed data obtained from pheromone traps were significant for all generations, with a tendency for correlation to decrease between observations separated by increasing replacement times of sticky inserts in delta traps. However, the sensitivity of visual estimates at low population levels was higher than estimates obtained from pheromone trap data. Mines counting provided a good estimate of the degree of moth infestation at low infestation levels [24].

**Figure 3:**
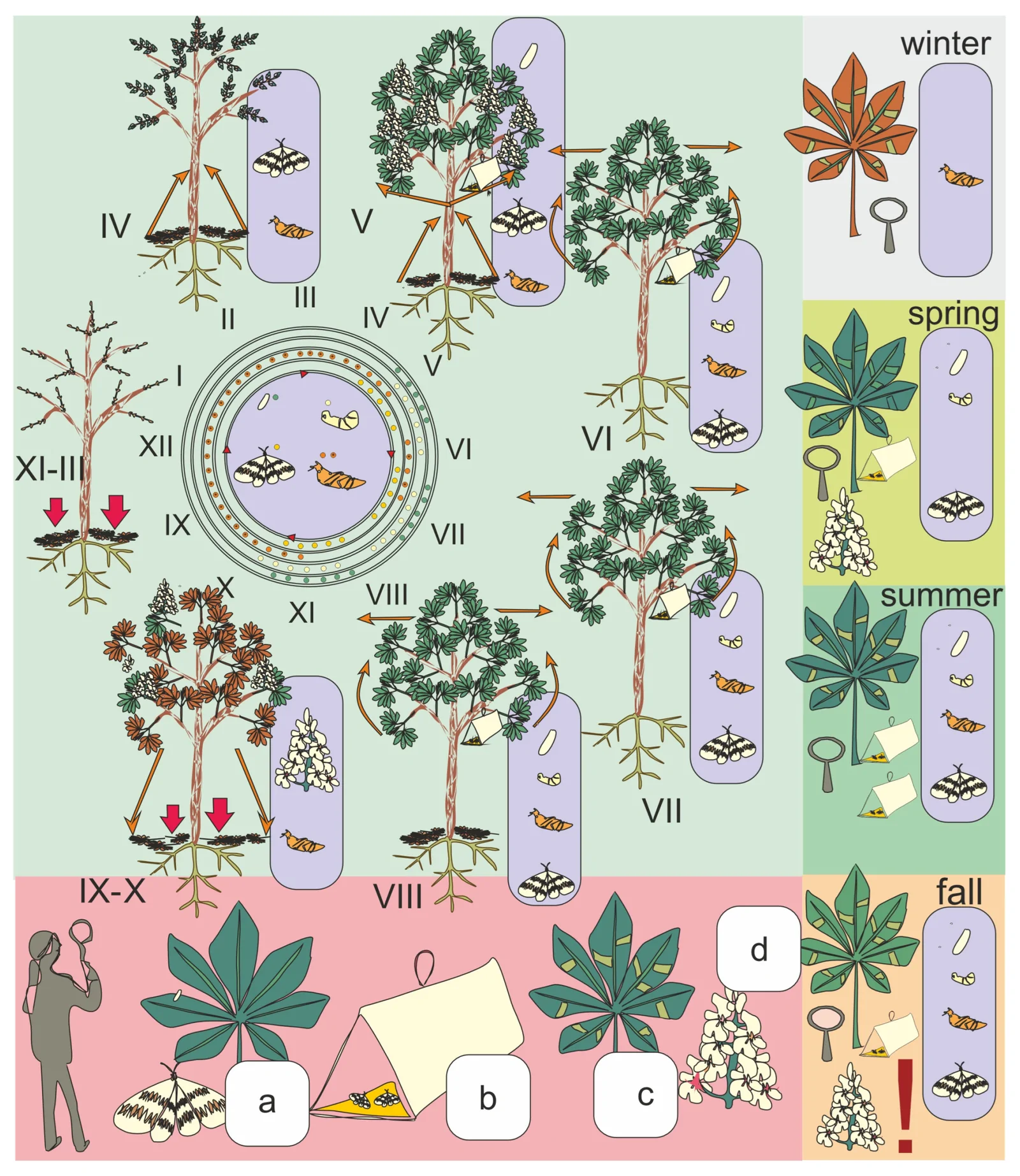
Integrated monitoring taking into account the life cycle (green background (**top**)) of horse chestnut plants and the leaf miner, indicating the life forms (in a cartouche, next to the tree) identified during monitoring of the *C. ohridella* population based on observations on selected leaves and using pheromone traps. The pie chart shows the months during which the corresponding phases of *C. ohridella* can be observed. April-May—pupae in last year’s litter and emergence of adults from pupae overwintering in the foliage in the trunk and skeletal branches; May (horse chestnut flowering), mass emergence—oviposition; May-June—development of all stages of the first generation of *C. ohridella* and the beginning of monitoring of male *C. ohridella* individuals using pheromone traps; July–August—flight and counting of subsequent generations of *C. ohridella*; September–October—the second flowering of weakened trees and the fall of leaves containing overwintering leaf miner pupae in cocoons, and the end of monitoring leaf mines and adults using pheromone traps. Visual assessment (pink background (**bottom**)) of the condition of pupae and adults; a—monitoring of egg laying and damage with mine counting; b—monitoring of flight intensity using traps; c—selection of leaves with characteristic mines and larvae of different ages; d—horse chestnut flowering, as an indicator of the onset of adults (spring time) and the dormancy breaking during severe tree exhaustion (re-blooming in autumn) as a result of metabolic disturbance caused by C. *ohridella*.

Figure 3 shows monitoring methods that include visual observation, the use of pheromone traps, and sampling of damaged leaves from litter or from the crowns of damaged trees.

Monitoring of parasitoids and parasitism levels is often assessed by means of opening mines and rearing insects in laboratory conditions. Thus, in the fstudy of Volter et al. (2022), mines were carefully cut from horse chestnut leaves and dissected under a stereomicroscope, after which the numbers of live, dead, and infected larvae and pupae were recorded [70]. Parasitic individuals, including dead pupae, were then placed individually in Petri dishes and examined for the presence of hatched parasitoids. In Germany, Freise and Heitland (2004) [9] analyzed tens of thousands of mines to determine the level of parasitism [28].

The effectiveness of the horse chestnut leaf miner parasitoid complex and their numbers were also determined in fallen leaves. Afterwards, parasitoids were released at different densities to study the dependence of parasitism rates on density and biological control of horse chestnut leaf miners using parasitoids [94].

**Figure 4:**
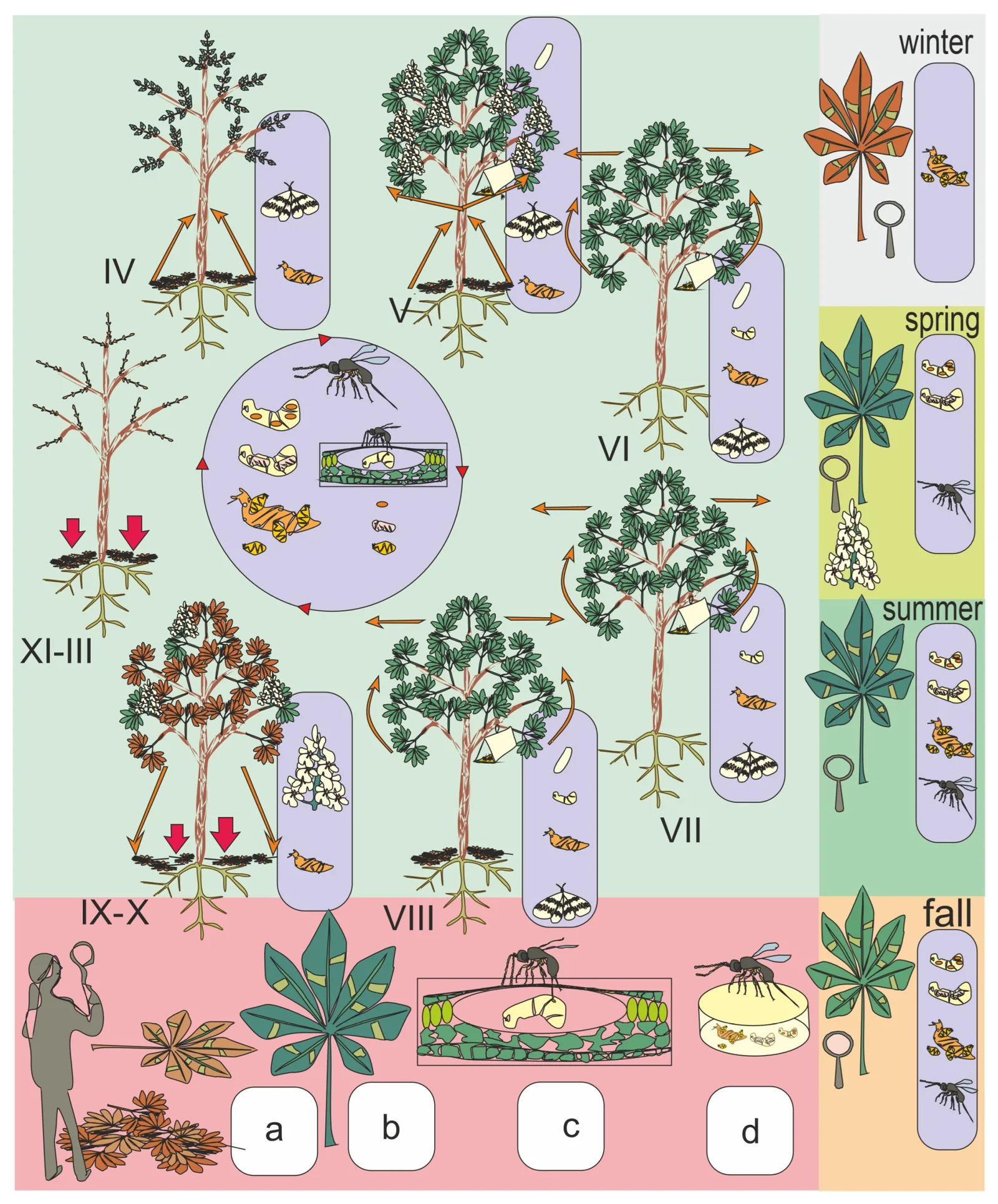
Monitoring of *C. ohridella* parasitoids in the framework of comprehensive monitoring of *C. ohridella* using visual monitoring and sampling in natural stands (green background (**top**)) taking into account life forms (in a cartouche: imago, egg, larvae of different ages, pupa, imago) by selecting damaged leaves, opening mines and laboratory rearing (Figure S1). April-May—assessment of emergence of imago *C. ohridella* and its parasitoids from pupae overwintering in foliage; May-June—search for parasitoid egg clutches on *C. ohridella* larvae and start of parasitoid monitoring; July–August—visual monitoring of eggs, larvae, pupae and imago parasitoids and sampling for laboratory testing, counting of larvae and pupae; September–October—leaf fall with overwintering leaf miner and parasitoids and end of monitoring. Visual assessment (pink background (**bottom**)): a—preparation of mines from collected leaves/litter; b—recording the number of live, dead and parasitized larvae and pupae collected during preparation of *C. ohridella* mines from damaged horse chestnut leaves; c—laying eggs by a parasitoid (wasp) into a *C. ohridella* larva from the leaf surface; d—placing *C. ohridella* pupae containing pupae of parasitic individuals into Petri dishes and adult emergence.

A scheme for tracking the life cycle of *C. ohridella* and monitoring parasitic insects on horse chestnut involves sampling fallen leaves or leaves damaged by leaf miners in the canopy of trees [103] and rearing infected larvae and pupae in insectaries in the laboratory, followed by identification of parasitic organisms and assessment of their harmfulness (Figure 4).

In different seasons and different months, the objects of monitoring of the horse chestnut leaf miner may vary and include eggs, larvae of different ages, pupae and adults.

**Figure 5:**
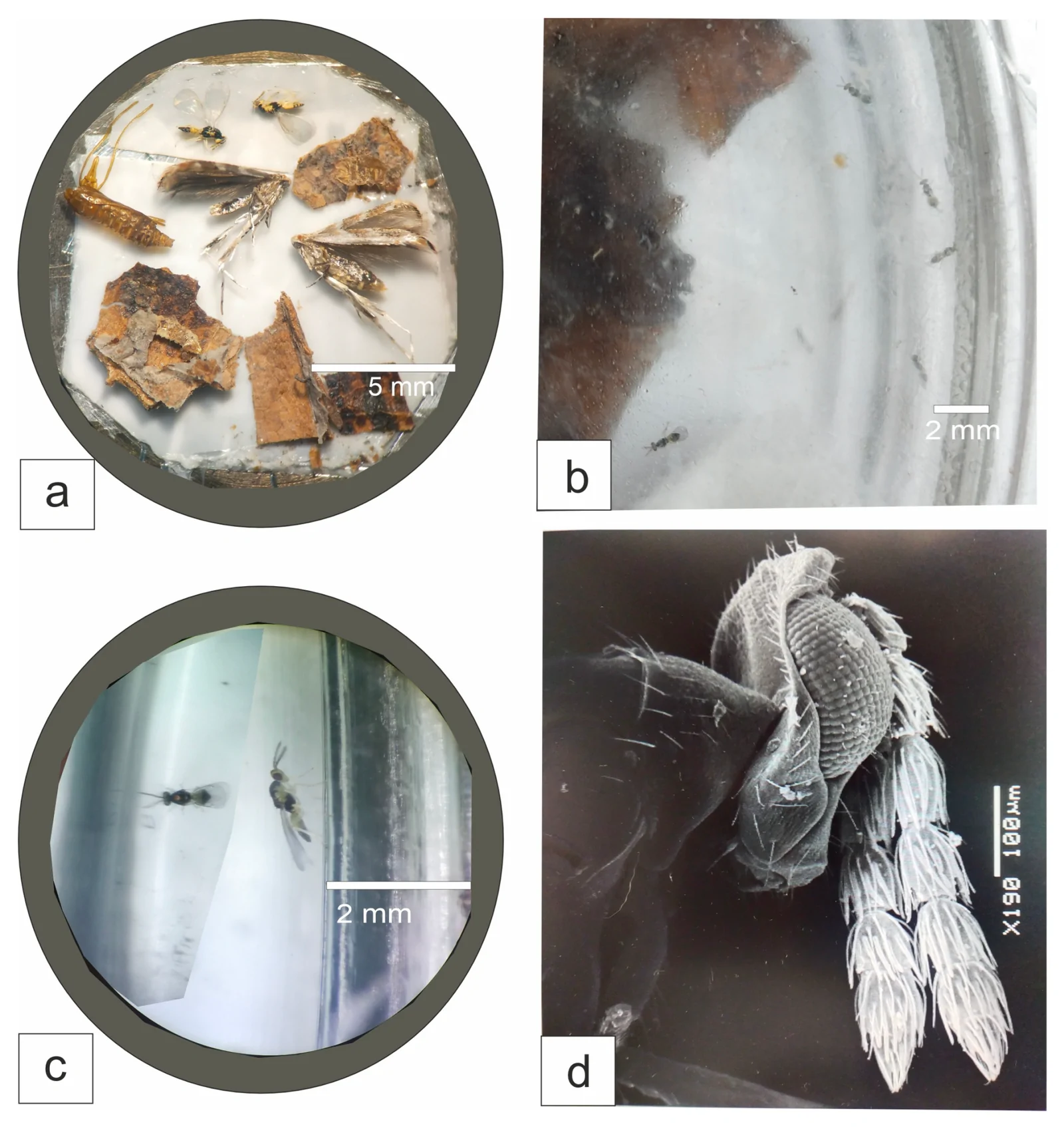
Samples of the horse-chestnut leaf miner and parasitoids reared in a Petri dish during laboratory cultivation and analysis. Key: (**a**) adult and pupa of *C. ohridella* and adult parasitic wasps reared from them; (**b**) rearing of pupae in excised mines and emergence of parasitic insects in a Petri dish; (**c**) dorsal and lateral views of reared adult wasps used for preliminary identification; (**d**) head of an adult parasitic wasp viewed under a scanning electron microscope following fixation, critical-point drying, and gold-palladium coating.

Monitoring of predators, parasitoids and pathogens involved in the regulation of the *C. ohridella* population consists of two interrelated stages: (1) recording the presence and establishing the number and monitoring the dynamics of damage caused by the pest (Tables S1 and S2). The presence of parasitoids is most clearly assessed and identified by rearing pupae from mines collected via leaf discs taken from the tree or from leaf litter (Figure 5).

Monitoring pests and parasitoids is perhaps one of the best ways to clearly determine the adaptation of native species that feed on invasive pests and establish that population regulation maintains a balance between plant viability, its ornamental qualities, and the development of pathogens caused by the invasive pest in the absence of natural regulatory mechanisms. This process is currently complex and not fully understood, but encouraging data indicate the formation of clear relationships based on the recognition and pairing of a parasitoid and a pest that is suitable for its ecological niche.

The effectiveness of predators, parasitoids, and pathogens is measured in conjunction with sample analysis when monitoring the development stages of the leaf miner moth *C. ohridella* but remains poorly understood and requires study and proper assessment of the interactions and role of each species capable of regulating population numbers. The role of ants capturing adults in the litter after winter remains particularly complex and unexplored. It cannot be ruled out that, with their activity, they are capable of destroying not only the moths themselves during their release from mines but also parasitoids, limiting their effectiveness (Table S3, Figure S3). Observations and detailed interaction studies and contributions of predators, parasitoids, and pathogens of the leaf miner *C. ohridella* as an important pest species and as a model object remain incomplete and require a transition to quantitative methods to identify the causes limiting natural population regulation and the potential for its occurrence in the future.

## 8. Conclusions

Although the use of parasitoids has the potential to be a method of biological control, a number of problems arise, particularly related to the problem of desynchronization of the development cycles of parasitoids and the horse chestnut leaf miner. One hypothesis that could explain this is that the area of origin (the beginning of the spread) of the leaf miner is in Southeast Asia. Polyphagous species may not attack *C. ohridella*, especially in the first generation, due to differences in their development cycles, since the phenology of parasitoids is not synchronized with the development of leaf miners in the spring, as shown in the work of Grabenweger, G. (2004) [63]. The low percentage of parasitism may also be due to the fact that parasitoids have a negative dependence on high host density.

Horse chestnut plantings, an introduced species, are located in parks, squares, alleys, and along boulevards, serving as decorative features in urban areas. These plantings are subject to high anthropogenic pressure and often have a limited number of other tree and shrub species. This limits the potential for stable interactions between different parasitoid species and the Ohrid leaf miner, a relatively recent phytophage. This can lead to significant variation in the number of affected individuals and the balance of biocenoses. Since the interactions between the introduced plant, the invasive phytophage, and various predators and parasitoids are relatively recent and unstable, a detailed study of this entire complex under the varied conditions of urban areas is required.

## Figures and Tables

**Table 1:** Parasitoids of *C. ohridella* in Russia and some European countries.

	Family	Country	Reference
*Adialytus ambiguus* (Haliday, 1834)	*Braconidae*	Russia	[75]
*Apanteles circumscriptus* (Nees, 1834)	*Braconidae*		
*Mesopolobus mediterraneus* (Mayr, 1903)	*Pteromalidae*		
*Eupelmus microzonus* (Forster, 1860)	*Eulophidae*		
*Scambus calobatus* (Gravenhorst, 1829)	*Ichneumonidae*		
*Achrysocharoides altilis* (Delucchi, 1954)	*Eulophidae*		
*A. atys* (Walker, 1839)	*Eulophidae*		
*A. butus* (Walker, 1839)	*Eulophidae*		
*A. cilla* (Walker,1839)	*Eulophidae*		
*A. niveipes* (Thomson, 1878)	*Eulophidae*		
*A. zwoelferi* (Delucchi, 1954)	*Eulophidae*		
*Baryscapus endemus* (Walker, 1839)	*Eulophidae*		
*Chrysocharis elongata* (Thomson, 1856)	*Eulophidae*		
*Ch. nautius* (Walker, 1846)	*Eulophidae*		
*Ch. prodice* (Walker, 1839)	*Eulophidae*		
*Ch. pubicornis* (Zetterstedt, 1838)	*Eulophidae*		
*Cirrospilus lyncus* (Walker)	*Eulophidae*		
*C. salatis* (Walker, 1838)	*Eulophidae*		
*C. staryi* (Boucek, 1959)	*Eulophidae*		
*Derostenus gemmeus* (Westwood, 1833)	*Eulophidae*		
*D. punctiscuta* (Thomson, 1878)	*Eulophidae*		
*D. minoeus* (Walker, 1838)	*Eulophidae*		
*Elachertus isadas* (Walker, 1839)	*Eulophidae*		
*Euderus albitarsis* (Zetterstedt, 1838)	*Eulophidae*		
*Hemiptarsenus wailesellae* (Nowicki, 1929)	*Eulophidae*		
*H. waterhousii* (Westwood, 1833)	*Eulophidae*		
*Closterocerus formosus* (Westwood, 1833)	*Eulophidae*		
*Pediobius alcaeus* (Walker, 1839)	*Eulophidae*		
*P. cassidae* (Erdos, 1958)	*Eulophidae*		
*P. facialis* (Giraud, 1863)	*Eulophidae*		
*Pediobius pyrgo* (Walker, 1839)	*Eulophidae*		
*Ratzeburgiola cristatus* (Ratzeburg, 1848)	*Eulophidae*		
*Sympiesis acalle* (Walker, 1848)	*Eulophidae*		
*S. dolichogaster* (Ashmead, 1888)	*Eulophidae*		
*Closterocerus trifasciatus* (Westwood)	*Eulophidae*	Czech Republic	[76]
		Germany	[77]
		Italy	[64]
		Russia	[75]
		Slovakia	[76]
		Slovenia	[76]
		UK	[78]
*Cirrospilus diallus* (Walker)	*Eulophidae*	Czech Republic	[76]
		Russia	[75]
		Slovakia	[76]
		Slovenia	[76]
*Cirrospilus talitzkii* (Boucek)	*Eulophidae*	Italy	[64]
*Cirrospilus variegatus* (Masi)	*Eulophidae*	Italy	[64]
*C. vittatus* (Walker)	*Eulophidae*	Czech Republic	[76]
		Germany	[77]
		Italy	[64]
		Russia	[75]
		Slovakia	[76]
		Slovenia	[76]
		UK	[78]
*Elachertus inunctus* (Nees)	*Eulophidae*	Germany	[77]
		Russia	[75]
*Pnigalio agraules* (Walker)	*Eulophidae*	Czech Republic	[76]
		Germany	[77]
		Italy	[64]
		Russia	[75]
		Slovakia	[76]
		Slovenia	[76]
		UK	[78]
*P. pectinicornis* (L.)	*Eulophidae*	Czech Republic	[76]
		Germany	[77]
		Italy	[64]
		Russia	[75]
		Slovakia	[76]
		Slovenia	[76]
*S. sericeicornis* (Nees)	*Eulophidae*	Germany	[77]
		Italy	[64]
		Russia	[75]
		UK	[78]
*Ch. nepherea* (Walker)	*Eulophidae*	Czech Republic	[76]
		Germany	[77]
		Russia	[75]
		Slovakia	[76]
		Slovenia	[76]
*Ch. nitetis* (Walker)	*Eulophidae*	Czech Republic	[76]
		Germany	[77]
		Russia	[75]
		Slovakia	[76]
		Slovenia	[76]
*Neochrysocharis chlorogaster* (Erdös)	*Eulophidae*	Germany	[77]
*Pediobius alcaeus* (Walker)	*Eulophidae*	Germany	[77]
		Russia	[75]
*P. saulius* (Walker)	*Eulophidae*	Czech Republic	[76]
		Germany	[77]
		Italy	[64]
		Russia	[75]
		Slovakia	[76]
		Slovenia	[76]
		UK	[78]
*Baryscapus nigroviolaceus* (Nees)	*Eulophidae*	Germany	[77]
		Russia	[75]
*Minotetrastichus frontalis* (Nees)	*Eulophidae*	Czech Republic	[76]
		Germany	[77]
		Italy	[64]
		Russia	[75]
		Slovakia	[76]
		Slovenia	[76]
		UK	[78]
*M. platanellus* (Mercet)	*Eulophidae*	Italy	[64]
		Russia	[75]
*Colastes braconius* (Haliday)	*Braconidae*	Czech Republic	[76]
		Germany	[77]
		Slovakia	[76]
		Slovenia	[76]
*Scambus annulatus* (Kiss)	*Ichneumonidae*	Czech Republic	[76]
		Germany	[77]
*Itoplectis alternans* (Gravenhorst)	*Ichneumonidae*	Czech Republic	[76]
		Germany	[77]

**Table 2:** Differences in the detection and study of *Cameraria ohridella* parasitoids across various regions of Europe.

Country	Parasitoids	Method for Assessing the Level of Parasitism	Level of Parasitism	Reference
Czech Republic	*Minotetrastichus frontalis*, almost 40% of contaminated mines, *Pnigalio agraules*, found in 19% of mines.	Laboratory	The overall level of parasitism was 0.98%	[76]
Slovakia	*Pediobius saulius* (75% parasitism) и *Minotetrastichus frontalis* (21% parasitism).	Laboratory	The overall level of parasitism was 11.34%	[76]
Slovenia	P. *saulius*, *Pediobius saulius* и *Minotetrastichus frontalis*.	Laboratory	Overall level of parasitism: 14.59%	[76]
UK	*Minotetrastichus frontalis* (Nees), *Pnigalio agraules* (Walker), *Sympiesis sericeicornis* (Nees), *Cirrospilus vittatus* (Walker), *Closterocerus*. sp., *M. frontalis* (22,1% parasitism)	Laboratory	The overall level of parasitism was 2.31%, and the mean level of parasitism was 2.20% (across all parks)	[78]
Czech Republic	*Pediobius saulius* (Walker), *Minotetrastichus frontalis* (Nees), *Closterocerus trifasciatus* and two unidentified species	Laboratory	The overall level of parasitism ranges from 1.9% to 20.5%, with a mean value of 7.2%	[70]
Greece, Bulgaria (natural plantings); Greece, Bulgaria, Croatia, Serbia, Macedonia (artificial plantations)	*Pediobius saulius*, *Minotetrastichus frontalis*, *Closterocerus trifasciatus*, *Chrysocharis nephereus* and *Cirrospilus viticola*, *Baryscapus nigroviolaceus*, *Colastes braconius*, *Chrysocharis phryn*, *Pteromalus semotus*, *C. phryne*, *C. braconius*	Laboratory	Parasitism rates were 12.0% in natural stands and 10.7% in artificial plantings	[72]
Switzerland	*M. frontalis*, *P. agraules*, *C. trifasciatus*, *C. nephereus*. Other species of parasitoids were encountered less frequently: *Colates braconius* (Haliday), *Sympiesis sericeicornis* (Nees). *C. nephereus*	Field/laboratory	The average level of parasitism was low, never reaching 5% at any stage, across various environmental conditions and generations	[19]
Russia	*Scambus brevicornis* Gravenhorst, *Pediobius alcaeus* (Walker), *Baryscapus nigroviolaceus* (Nees), *Sympiesis acalle* (Walker, 1848)		The level of parasitism ranged from 12.0% to 33.6%	[74]

## Data Availability

The data are presented in the text of the review.

## Supplementary Materials

The following supporting information can be downloaded at: https://www.mdpi.com/article/10.3390/biology15201854/s1, Figure S1: Larvae; Method for fixation of different stages of development of *C. ohridella*; Figure S2: Estimation of *C. ohridella* spread on horse chestnuts; Table S1: Monitoring of *C. ohridella* damage to common horse chestnut; Table S2: Recording the presence of organisms (and traces of their activity) that are potentially capable of limiting the development and reproduction of *C. ohridella*; Figure S3: Enemies of *C. ohridella*; Table S3: Current state of interaction studies and contributions predators, parasitoids, and pathogens of leaf miner *C. ohridella*.
