# Supplementary material for: Native Entomophages in the Regulation Cameraria ohridella Deschka & Dimic Populations: Potential and Limitations for Control
Source: Biology (Basel). 2026 Oct 15;15(20):1854. doi: 10.3390/biology15201854 (PMC13701256; doi:10.3390/biology15201854)
Supplement: Supplementary file 1 [file biology-15-01854-s001.zip › biology-4530034-supplementary.pdf]

Supplementary Materials

Review "Monitoring and control of Cameraria ohridella Deschka & Dimic populations by native entomophagous species"

Liliya R. Bogoutdinova, Ekaterina M. Savenko, Alexander A. Gulevich, Ekaterina N. Baranova

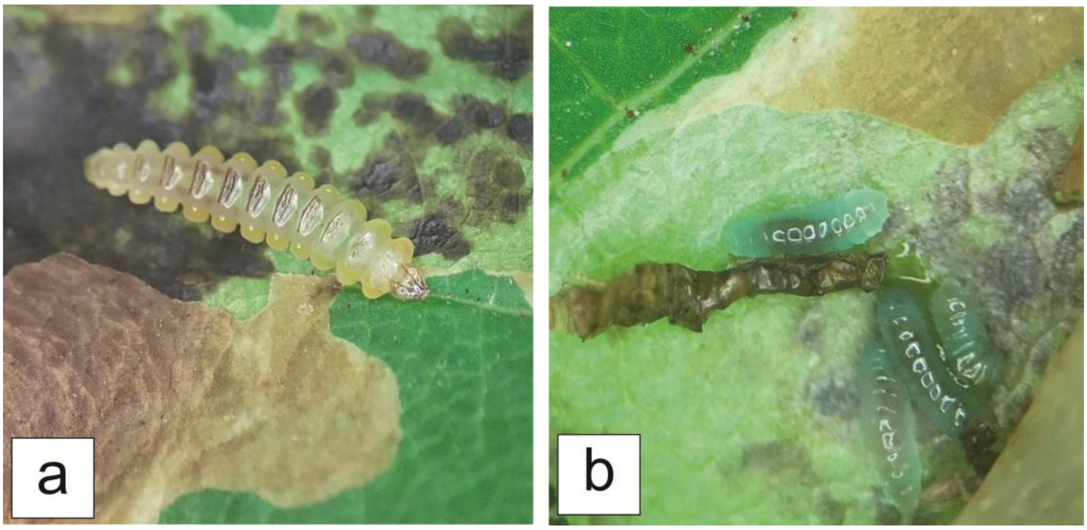

Figure S1. Larvae. Abbreviations: a—*C. ohridella* larva in an *A. hippocastanum* mine on a leaf from the *Aesculus* species collection on the territory of the Main Botanical Garden of the Russian Academy of Sciences in Moscow (imago); b—larva of a parasitic wasp of the family Eulophidae in a *C. ohridella* mine on a horse chestnut leaf.

Method for fixation of different stages of development of *C. ohridella*

Insects were fixed during the day at +4 °C in a 2.5% solution of glutaraldehyde (Merck, Germany) prepared in 0.1 M phosphate buffer (pH is 7.2) with the addition of 1.5% sucrose. Then, the samples were dehydrated at +4 °C for 30 min in each alcohol solution with successively increasing concentrations: 30%, 50%, 70%, 96%, and in three changes of absolute ethanol. After that, the samples were transferred to liquid CO2 under pressure in the device for drying at the "critical point" and slowly heated under pressure. When the pressure and temperature together passed the so-called "critical point" (31 °C and 74 bar), the pressure was reduced, thus drying the samples without any damage. Next, a thin (from 1 nm to more) metal layer was deposited onto the samples to enhance conductivity and add mechanical strength to the sample (sputtering unit SPI supplies, SPI, Santa-Clara, CA, USA). The photographs were obtained using a JSM-6380LA scanning electron microscope (JEOL Ltd., Tokyo, Japan).

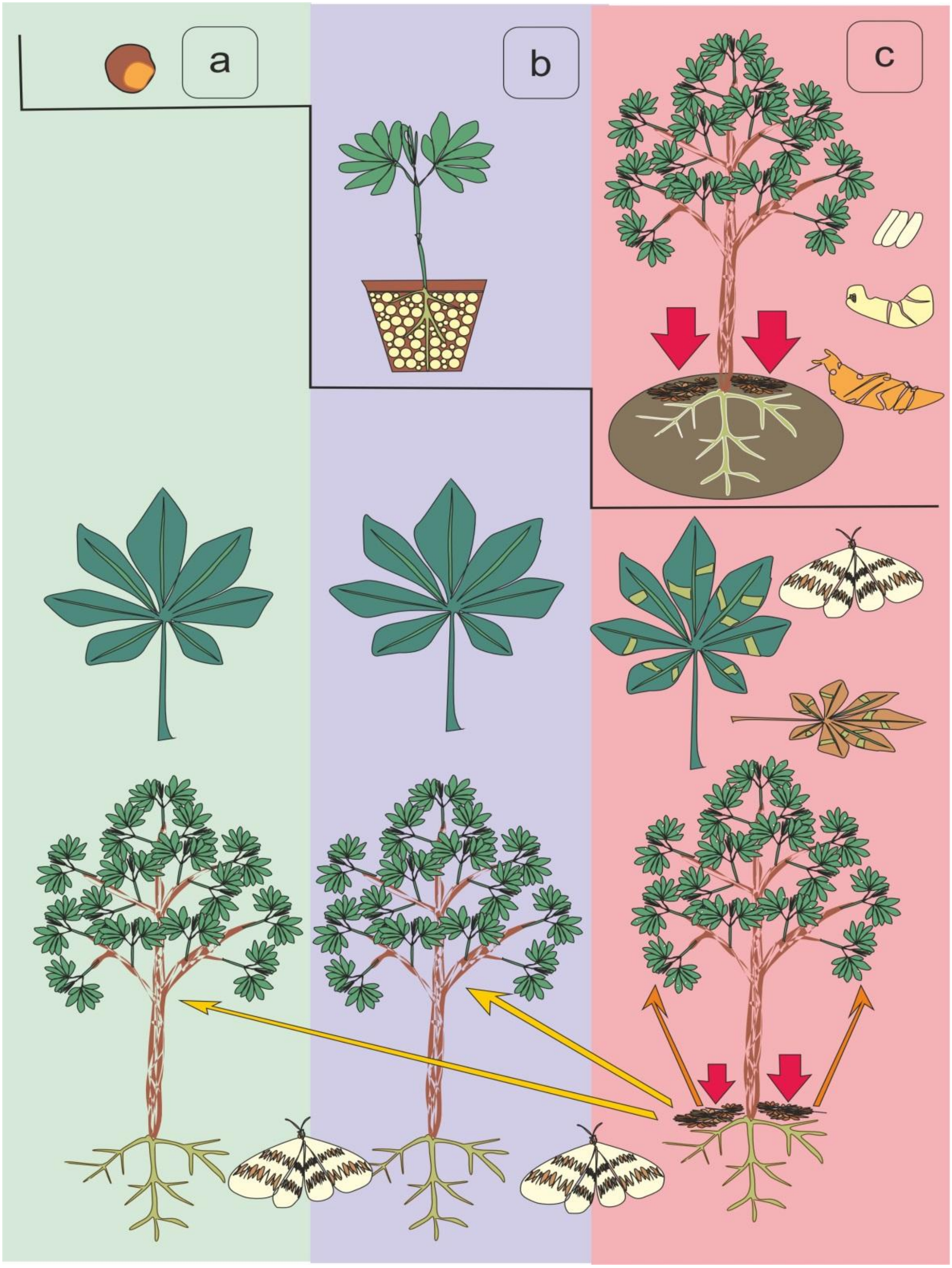

Figure S2. Estimation of *C. ohridella* spread on horse chestnuts depending on the method of introduction: green background – by seeds, blue background – by seedlings grown in a substrate, transported without plant debris; c – with seedlings or large-sized material, often accompanied by the transport of pupae, eggs, and, less often, larvae and imago. For this reason, it is the use of large-sized plants that most often leads to the spread of invasive pests. The development of the *C. ohridella* emergence process can be assessed by monitoring both plants grown from seeds (a) and plants brought in clean substrate (b). The developmental stage of *C. ohridella* during transport of large-sized plants is unimportant, as adults are least likely to be transported with large trees (c). However, they frequently cause damage to plants planted from seeds or young plants transported bare-root or in sterile substrate. Eggs, larvae, and pupae, unlike adults, are often transported on large plants in plant debris, on the surface, or within mines (c).

Table S1. Monitoring of *C. ohridella* damage to common horse chestnut.

№	Control of the development processes of *C. ohridella*	Month of accounting
1	Sampling in tree growth areas (egg, adult, larvae, pupae)	I-XII

1.1	Trunk, skeletal branches after the first emergence of adults	IV-V
1.2	Damaged leaves and mines on leaves in the crown	V-IX
1.3	Fallen leaves and their fragments with cocoons and mines	X-III (IV)
2	Control of the total pest population *C. ohridella*	I-XII
2.1	Determination of population size by the number of captured males using pheromone traps	V-VIII
2.2	Counting mines, larvae and pupae from a random sample of leaves in the crown	V-IX
2.3	Accounting for population dynamics during the vegetation season and at the end of the season	V-IX

Table S2. Recording the presence of organisms (and traces of their activity) that are potentially capable of limiting the development and reproduction of *C. ohridella*.

Object of observation	Visual inspection of horse chestnut growth sites, recording of detection, establishing the fact of interaction	Identification of secondary indicators of species involvement that are potentially capable of regulating abundance
C. ohridella	Presence/absence, quantity, stage	Reduction in numbers due to the impact of natural enemies, determined by the presence of empty mines, damage to larvae, pupae or adults
Birds (great tit, blue tit, nuthatch, common treecreeper, house sparrow, tree sparrow)	Presence/absence of birds in the crown, on the ground surface under the crown, birds singing, number of individuals, active preying on larvae, pupae, and moths	Traces of excrement, presence of damaged mines, traces of scratches on the surface of leaves, empty mines
Garden and forest ants	Presence/absence of individuals on the trunk, branches, soil, leaf surface, ground, quantity, active foraging of the moth, collection and transfer of available larvae and pupae from open or bird-damaged or destroyed mines	Empty mines in the litter, empty mines on the leaves in the crown
Parasitoids (*Minotetrastichus frontalis, Pnigalio nemati, Pediobius saulius, Braconidae*)	Presence/absence of individuals on trunks, leaves, number of damaged pupae or larvae depending on the species	No emergence of imago, presence of several pupae in the one mine
Entomopathogenic micromycetes (*Beauveria bassiana, Paecilomyces fumosoroseus / Isaria fumosorosea, Metarhizium robertsii / anisopliae)*	Presence/absence of emergence, number of damaged pupae or larvae depending on the characteristic pathogenesis (inside the mine, in litter)	No emergence, change in color of the mine when held up to the light, change in color of the chitinous shell of the larva or pupa

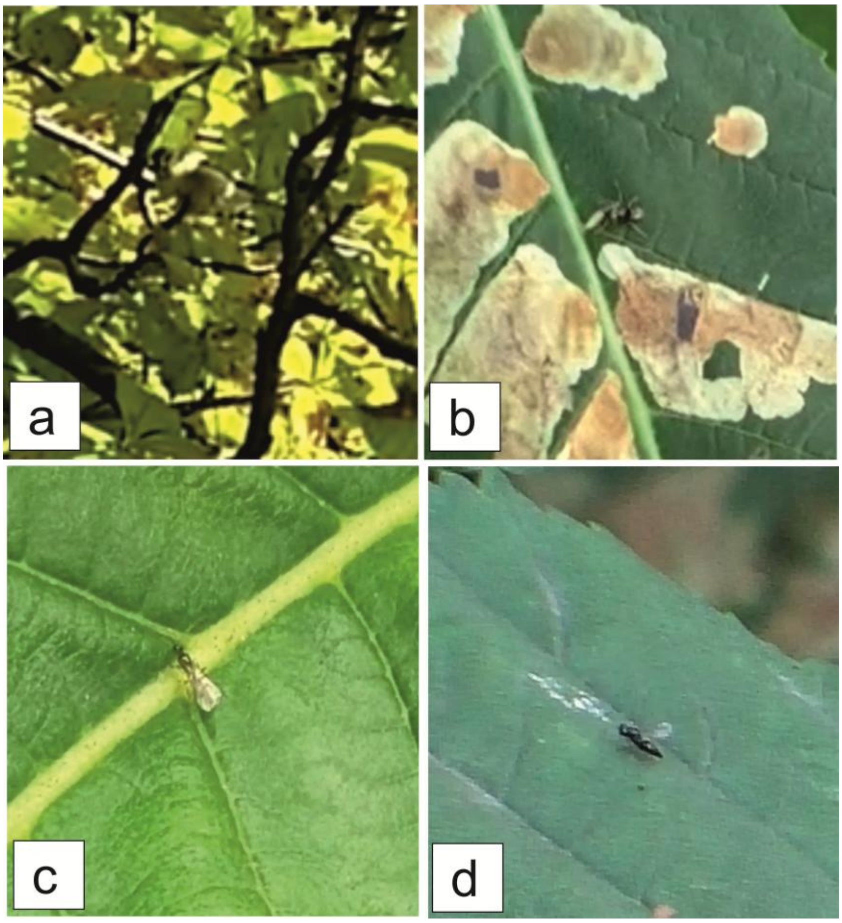

Figure S3. Abbreviations: a – tit of the genus *Parus*; b – ant of the genus *Lasius* with a *C. ohridella* larva; c, d – parasitic wasps of the family *Eulophidae*.

Table S3. Current state of interaction studies and contributions predators, parasitoids, and pathogens of leaf miner *C. ohridella*

№	% general contribution to the elimination of leaf miner	% contribution to the elimination of leaf miner	
Availability	100	100	No damage to foliage healthy plants
	4 – 90%	4–13% (mild winters)<br>Up to 90%	Empty cocoons and mines indicate the presence of predators (formica)[49,50]
	1,9 – 20,5%	from 1.9% to 20.5%	The presence of both large and small pupae indicates the presence of parasitoids [90]
	5– 30%	5% to 12% (20–30% - lab experiment)	The presence of a non-motile larva or pupa with an uncharacteristic color of the chitinous shell and/or covered with a thin layer of hyphae indicates the presence of fungal pathogens [60]
Efficiency	sporadic observations	Studied*	Complete absence of larvae, pupae or adults in collected leaf samples, litter and visual observations [13]
	sporadic observations	Studied*	The ratio of empty cocoons/min, larvae of different ages and pupae to the total number of imagoes, pupae and larvae [86]
Strategy	no research data	Studied*<br>Not used**	Visual inspection and determination of involvement (active process) [97]
	no research data	Studied*<br>Not used**	Creation of conditions for active assessment (placing samples of affected leaves, mines with larvae, pupae on the ground or in the crown of a tree with subsequent observation (with mandatory subsequent disposal)[28]
	no research data	Not studied***<br>Not used**	Recognition testing, imitation training (placement of exposed mines, larvae, pupae, imago to observe the reaction taking into account the speed of reaction)[73]

* - sporadic observations; ** - no research data; ***- not studied.
